# Application of PIXE for Tear Analysis: Impact of Mineral Supplementation on Iron and Magnesium Levels in Athletes

**DOI:** 10.3390/nu17122010

**Published:** 2025-06-16

**Authors:** Tal Zobok, Yulia Sheinfeld, Basel Obied, Yoav Vardizer, Alon Zahavi, Yakov Rabinovich, Olga Girshevitz, Nahum Shabi, Dror Fixler, Nitza Goldenberg-Cohen

**Affiliations:** 1 Faculty of Medicine, Hadassah Hebrew University Medical Center, Jerusalem 91120, Israel; tal331995@gmail.com; 2 Department of Ophthalmology, Bnai-Zion Medical Center, Haifa 339419, Israel; sheinfel@walla.co.il (Y.S.); basel.obied01@gmail.com (B.O.); yoavardizer@gmail.com (Y.V.); yakov.rabinovich@gmail.com (Y.R.); 3 Department of Ophthalmology, Rabin Medical Center, Beilinson Hospital, Petach Tikva 4941492, Israel; alonzahavi@gmail.com; 4 Faculty of Medicine, Tel Aviv University, Tel Aviv 6997801, Israel; 5 Laboratory of Eye Research, Felsenstein Medical Research Center, Petach Tikva 4941492, Israel; 6 Institute of Nanotechnology & Advanced Materials, Bar Ilan University, Ramat Gan 5290002, Israel; olga.girshevitz@biu.ac.il (O.G.); nahum.shabi@biu.ac.il (N.S.); dror.fixler@biu.ac.il (D.F.); 7 The Krieger Eye Research Laboratory, Bruce and Ruth Faculty of Medicine, Technion—Institute of Technology, Haifa 3498838, Israel

**Keywords:** dietary supplements, fitness, nutrition, particle induced X-ray emission (PIXE), sports, tears

## Abstract

**Background/Objectives**: To evaluate the concentrations of trace elements in tear fluid among athletes using particle-induced X-ray emission (PIXE), and to assess the associations with gender, sports intensity, and nutritional supplement intake. **Methods**: In this cohort study, 84 athletes engaged in high- or low-intensity sports completed a demographic and supplement-use questionnaire. Tear samples were collected using Schirmer strips and analyzed for elemental composition with PIXE, a high-sensitivity technique suited for small biological samples. Multivariate and nonparametric statistical analyses were used to compare groups. **Results**: There were 46 males and 38 females, aged 17–63 years (mean 30.21 years). Tear phosphorus, potassium, and sulfur concentrations were higher in women than men and higher in women participating in low-intensity compared to high-intensity sports. Tear concentrations of magnesium were higher in men participating in high-intensity sports compared to low-intensity sports. They were higher in men than women regardless of supplement intake. Iron concentrations were higher in men than women only when neither group was taking supplements. Smoking had a slight inverse relationship to iron values. Iron levels were particularly high in men participating in intense sports and low in smokers. Magnesium supplements were associated with raised magnesium levels in tears. **Conclusions**: This study demonstrates an association between trace element levels in human tears and gender, sports intensity, and food supplement intake. PIXE enables the evaluation of trace element concentration in tears, which may serve as potential biomarkers for the clinical assessment of athletes’ health.

## 1. Introduction

Physical activity has been shown to be associated with health-related quality of life [1]. Participation in sports is often associated with changes in diet and consumption of nutritional supplements [1]. Athletes of all levels (extreme, competitive, amateurs) may use dietary supplements to improve performance, build muscle, and increase stamina. Intense exercise can cause substantial metabolic stress and consequent accumulation of metabolites (e.g., lactate, hydrogen ions) and increased markers of muscle damage and muscle fatigue [2]. In this setting, supplements are intended to counteract vitamin and mineral deficiencies, often due to limited food intake by athletes, and modify the adaptive cellular signaling response through multiple mechanisms [3]. Dietary supplements are marketed in various forms, including tablets, powders, snacks, and drinks. The most common ones contain proteins, amino acids, creatine, caffeine, and fish oil [3], and to a lesser extent, minerals such as iron, magnesium, zinc, and various vitamins. Antioxidants have been recommended on the assumption that they help raise the body’s defenses against the production of free radicals induced by physical activity, thereby maintaining normal enzyme function and preventing oxidative stress [3]. Dietary supplements containing vitamins E, A, and C, selenium, succinic and alpha-lipoic acid, coenzyme Q10, glutathione, and other natural sources of antioxidants might help to postpone fatigue and improve physical efficiency [3]. However, interventional data on dietary and supplement strategies are limited, and the few published studies so far had several critical methodological flaws, precluding solid conclusions on efficacy [1,2,3]. Even less is known about the effect of continued supplementation concurrent to sustained, cumulative oxidative stress during long-term endurance training programs.

The tear film is composed of three distinct layers—lipid, aqueous, and mucin—protecting the ocular surface. It is distilled from blood plasma, and its composition can reflect changes in other body fluids and organs [4,5]. Sampling tears is a relatively easy, noninvasive procedure requiring minimal manipulation [6]. The ability to accurately analyze trace elements in tear film was shown in a study of healthy subjects from urban and rural areas [7]. Others reported an association of tear fluid concentrations of aluminum with ingestion of food in a communal dining room that used aluminum pots [8], indicating that tear content is affected by nutritional intake. However, few studies have measured the levels and variability of trace elements in tears or investigated their association with intake of nutritional supplements [7] or their correlation with levels in blood, sweat, or urine [9]. One group measured the effect of disease (diabetes) on trace elements in tears, with limited accuracy [10]. Whether tear film may serve as a reliable biomarker in persons exposed to high concentrations of trace elements due to intake of nutritional supplements or environmental exposure remains unknown [7].

Determining the concentrations of electrolytes in tears is difficult because of the small sample volumes, up to 10–20 µL [11], and lack of reliable analytic techniques. Methods applied in studies of metallic trace elements in tears in animals [11] included atomic-absorption spectrometry, atomic-emission spectrometry with a direct current or inductively coupled plasma, anodic stripping voltammetry, neutron activation analysis, and gas chromatography [11]. Matysik and Werner [12], using anodic stripping voltammetry in a capillary flow injection system, found low tear levels of lead, cadmium, and copper (µg/L).

Particle-induced X-ray emission (PIXE) is a technique for the determination of the elemental composition of a material. It is highly sensitive and can analyze even minimal volumes with high accuracy [8]. Studies have suggested that PIXE may be amenable for use in tear analysis [8]. The aim of the present study was to apply PIXE to analyze the concentrations of trace elements in tear fluid of athletes. The effects of gender, intensity of activity, and intake of supplements were assessed.

While previous studies have commonly used blood or urine to assess mineral levels, these methods are invasive and may be affected by acute physiological changes. Tear fluid, derived from blood plasma, is a non-invasive and easily accessible medium that may reflect systemic metabolic alterations, including those related to exercise and nutritional supplementation. Despite its potential, tear-based trace element analysis remains underexplored. In this context, the use of PIXE offers a sensitive and accurate technique for detecting such elements in small tear samples. To our knowledge, this is the first study to apply PIXE analysis to evaluate trace element concentrations in the tears of athletes, exploring potential associations with physical activity intensity and supplement use. The novelty of this study lies in applying PIXE to investigate whether tear fluid can serve as a surrogate marker for systemic elemental status in athletes, potentially paving the way for future non-invasive monitoring tools in sports medicine.

## 2. Materials and Methods

This study was approved by the Institutional Review Board of Bnai Zion Medical Center (Protocol No. 0117-19 BNZ, approved 17 May 2020) and conducted in accordance with the Declaration of Helsinki. The study participant recruitment started on 2 April 2021 and ended on 30 August 2021. All participants provided written informed consent before enrollment.

The cohort included 84 athletes attending three urban fitness centers representing different training intensities: a boxing gym (31 men, 4 women), a CrossFit gym (6 men, 1 woman), and a studio-based exercise gym (9 men, 33 women). All adult attendees present during the study period were invited to participate. The study cohort reflects real-world participation patterns in gym-based sports activities, where women were notably underrepresented in high-intensity training settings such as boxing and CrossFit. As such, the subgroup distribution was inherently unbalanced.

All adult attendees of three urban gyms were eligible to participate and were invited during the recruitment period. Inclusion was limited to consenting adults aged 18 years and older who were regularly engaged in physical training at one of the study gyms. Participants were excluded if they had any of the following: contact lens use, diagnosed glaucoma or chronic ocular medication use, dry eye syndrome or anterior segment pathology, recent swimming activity (within 2 h), or cosmetic application around the eyes (e.g., fresh eyeliner or mascara).

Each participant completed a standardized questionnaire covering demographic data, training frequency and intensity, supplement use, and smoking status (Table 1), and provided a tear sample. Variables were statistically compared between groups.

Tear samples were collected from all participants using color-free TearFlo sterile tear flow test strips, also known as Schirmer filter papers (Madhu Instruments, New Delhi, India) [12]. In brief, the filter paper was placed at the lower fornix for 1 min. Consequently, approximately a 5 mm section was moistened by tears. It is important to note that in a conventional PIXE measurement, only a small portion of the sample can be measured, limited by the size of the beam collimator (1.5 mm in diameter). The sample of each subject was stored in a separate Eppendorf plastic tube (Merck, Herzeliya, Israel). Topical anesthetic drops or gloves were not used, to avoid possible contamination.

PIXE analysis was performed with a 1.7 MV Pelletron Accelerator (National Electrostatics Corp., Middleton, WI, USA) at the Bar Ilan Institute of Advanced Materials using a proton beam of 2.017 ± 1 Kev MeV, as previously described [12]. Specifications were beam current ~11 nA, nominal diameter 1.5 mm, and integrated charge (Q) 3 µC. The normal incident beam was used in all measurements. PIXE spectra were collected with the Super Silicon Drift Detector (Fast SDD C2(70), Amptek Inc., Bedford, MA, USA). The detector was positioned at 40° to the beam normal (IBM geometry) with a solid angle of 4.1 msr. A 12 µm Mylar filter coated with 200 nm of tantalum was utilized to reduce background in the low-energy area. PIXE spectra were processed with GUPIX software, version 2.2.4, on the assumption that targets were thin and homogeneous and that all elements were in non-oxide form. Samples were mounted on the holder using double-sided, self-adhesive carbon tape. As PIXE measurements were performed in a high-vacuum chamber (10^−7^ Torr), all samples were dried before examination [13].

The use of PIXE for analyzing trace elements in tear samples was based on prior validation studies demonstrating its sensitivity, accuracy, and reproducibility for small-volume biological samples. Calibration procedures were conducted using multi-elemental standard solutions on dried matrix spots, as detailed in Krmpotić et al. [14]. Our group has successfully applied this method in prior human and animal studies involving tears, blood, and tissue, supporting its suitability for this application.

Quantitative variables were summarized by median, after reduction of the subject under detection and interquartile ranges. To test the association between two categorical variables, χ^2^ test or Fisher’s exact test was used. Quantitative variables were compared between two independent groups with the nonparametric Mann–Whitney test and among three independent groups with the nonparametric Kruskal–Wallis test. Bonferroni correction of the significance level was applied to multiple pairwise comparisons. Spearman nonparametric correlation coefficient was calculated to determine the strength of the association between two quantitative variables. Nonparametric tests were used because of the non-normal distribution of the variables compared. All tests were two-tailed. A *p*-value of 0.05 or less was considered significant.

To further assess the effects of gender, sport intensity, and nutritional supplements on tear trace element composition, multivariate statistical analysis was performed using MetaboAnalyst version 6.0. To address values below the limit of detection (LOD), elements with less than 50% valid data (Cr, Cu, and Fe) were excluded from the analysis. For the remaining elements, missing values were imputed using half of the LOD value, under the assumption that undetected measurements reflect concentrations below the detection threshold. Missing values were not excluded entirely due to the relatively small sample size. Data preprocessing included normalization to the total sum, followed by log transformation and standardization to zero mean and unit variance. Partial Least Squares Discriminant Analysis (PLS-DA) was performed to explore multivariate relationships and identify variables contributing to group separation. Variable Importance in Projection (VIP) scores were calculated to assess the contribution of each variable, with scores ≥ 1 indicating substantial influence in discrimination. A biplot was generated to visualize the sample distribution and variable loadings. Statistical significance of group separations was assessed using Permutational Multivariate Analysis of Variance (PERMANOVA) with 999 permutations. *p*-values < 0.05 were considered statistically significant.

## 3. Results

### 3.1. Study Participants

The cohort included 84 subjects, of whom 42 were attending a boxing or CrossFit gym (high-intensity sports) and 42 a studio-based gym (low-intensity sports). Mean age was 32.9 ± 11.2 years (median 32 years), and mean weight was 74.4 ± 16 kg (median 72 kg). Mean weekly duration of exercise activity was 6.4 h (median 5 h, range 2–25 h). The questionnaire-derived data on demographics, intensity of sports activity, supplement intake, and smoking status are summarized in Table 1.

### 3.2. PIXE Analysis of Trace Elements in Tears

#### Gender Differences

Findings on PIXE analysis of the elemental concentrations of tears in the cohort are summarized in Table 2.

### 3.3. Potassium, Phosphorus, Sulfur

**Figure 1:**
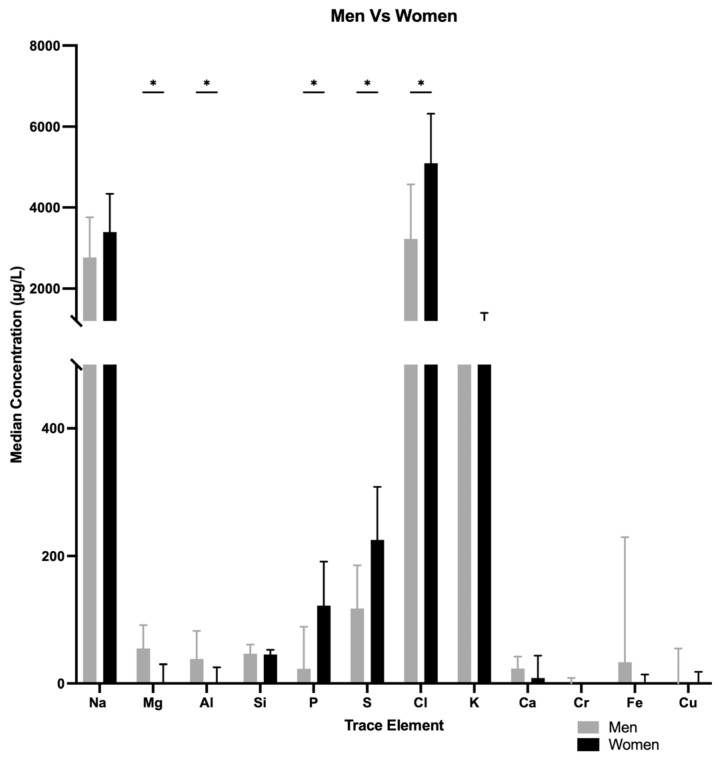
Median concentration differences in various trace elements when comparing men vs. women. * *p* < 0.05.

Median concentrations of potassium (K), sulfur (S) and phosphorus (P) were higher in tears of women than men (K, 1117 µg/L vs. 651.4 µg/L, *p* = 0.000; S, 240.95 µg/L vs. 123.75 µg/L, *p* = 0.001; P, 122.8 µg/L vs. 28.135 µg/L, *p* = 0.000) (Figure 1).

**Figure 2:**
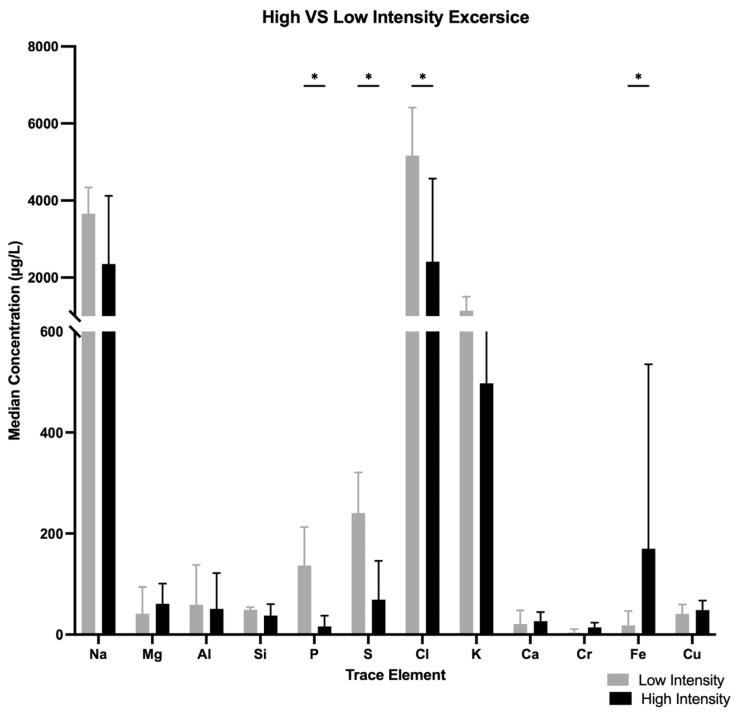
Median concentration differences of various trace elements when comparing high- vs. low-intensity exercises. * *p* < 0.05.

Median concentrations of K, S and P were lower in women participating in high-intensity sports (boxing, CrossFit) than in women attending studio-based gyms, K, 519.8 [148.35–907.1] µg/L vs. 1145.5 [943.07–1506] µg/L, *p* = 0.002; S, 91.96 [17.63–152] µg/L vs. 252.75 [170.7–331.57] µg/L, *p* = 0.005; P, 17.76 [8.894–46.905] µg/L vs. 136.85 [93.5–212.925] µg/L, *p* = 0.000) (Figure 2).

Median concentration of Fe were higher in high-intensity exercise than lower-intensity exercise (188 [108.2–556.75] µg/L vs. 18.32 [3.708–46.765] µg/L, *p* = 0.000).

### 3.4. Iron

**Figure 3:**
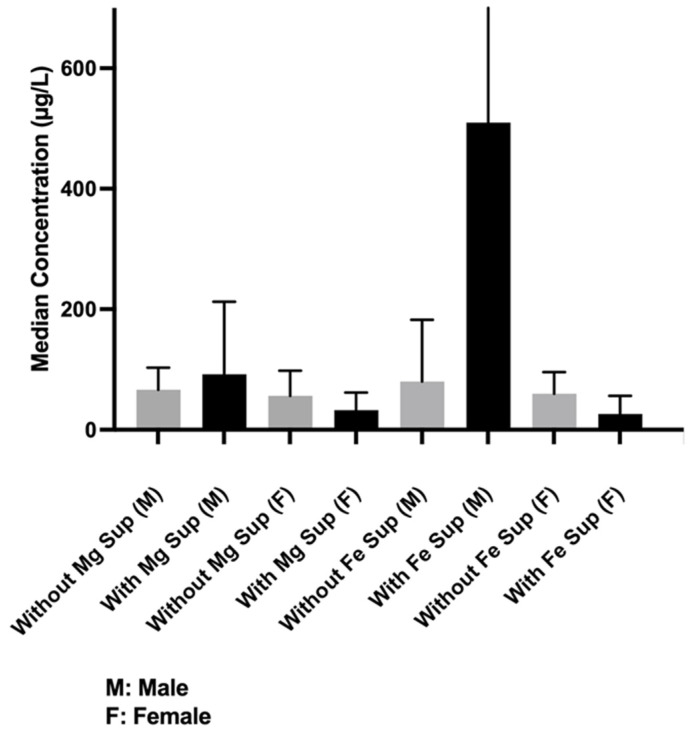
Median concentration differences of iron (Fe) when comparing participants (men and women) with and without supplements, men only with and without supplement, or women only with and without supplements.

Among subjects who did not take iron (Fe) supplements, men had a higher median concentration of Fe in tears than women, 58.21 [34.7475–92.287] µg/L vs. 45.655 [30.17–94.83] µg/L, *p* < 0.0109) (Figure 3). Among subjects taking Fe supplements, the difference between the genders also achieved statistical significance (men, 323.7 [123.925–810.875] µg/L, women, 18.22 [1.69–59.39] µg/L, *p* < 0.002). However, the difference in median Fe concentration between men who did not take Fe supplements and women who took supplements was not statistically significant (58.21 [34.74–92.8] g/L vs. 18.22 [1.69–59.39] µg/L, respectively, *p* = 0.1775).

Men who were taking Fe supplements had a relatively higher, albeit nonsignificant, median Fe concentration than men who were not (323.7 [123.925–810.875] µg/L vs. 58.21 [34.74–57.54] µg/L, *p* = 0.809). Women who were taking supplements had a lower, albeit nonsignificant, mean Fe concentration than women who were not (18.22 [1.69–59.39] µg/L vs. 45.655 [30.17–94.83] µg/L, *p* = 0.280) (Figure 3).

### 3.5. Magnesium

Men had a higher median magnesium (Mg) concentration than women with and without Mg supplement (with: 60.715 [39.09–102.3] µg/L vs. 48.49 [39.74–57.25] µg/L, *p* = 0.01. without: 59.07 [36.7–97.88] µg/L vs. 45.655 [25.225–97.88] µg/L, *p* = 0.014). Overall, subjects who took Mg supplements had higher-than-normal Mg concentrations in tears regardless of the level of sports activity. However, the difference from subjects who did not take Mg supplements did not reach statistical significance (59.07 [36.7–94.26] µg/L vs. 58.35 [31.84–94] µg/L, *p* = 0.137) (Figure 3).

### 3.6. Differences by Level of Sports Intensity

#### 3.6.1. Iron

Median Fe concentrations in tears of men participating in high-intensity sports were 107.1 [0–966] µg/L (boxing) and 76.55 [0–1637] µg/L (CrossFit), and not detected in men participating in studio-based sports. The difference between the whole high-intensity sports group (boxing + CrossFit) and low-intensity sports group was statistically insignificant (*p* = 0.081), regardless of Fe supplement intake (Figure 2). The difference remained statistically insignificant on comparison only of male CrossFit trainers with males training in studio-based gyms (*p* = 0.097). There were no significant differences between males training in CrossFit and boxing gyms.

The effect of age was negligible. Smokers showed a trend for reduced concentrations of Fe regardless of sports activity level (*p* = 0.068).

#### 3.6.2. Magnesium

Median Mg concentrations in tears of men engaged in high-intensity sports were 59.7 [0–555.4] µg/L (boxing) and 48.29 [7.03–1154] µg/L (CrossFit), and undetected for men training in studio-based gyms. The difference between the whole high-intensity sports group (boxing + CrossFit) and the low-intensity sports group was statistically significant (*p* = 0.0050) (Figure 2). The difference remained significant in comparison of each subgroup of high-intensity athletes (boxing, CrossFit separately) with the low-intensity sports group (for CrossFit, *p* = 0.013; for boxing, *p* = 0.002). There were no significant differences between the two high-intensity sports subgroups. The effect of age was negligible.

Mg concentrations in athletes who consumed Mg supplements were higher than in athletes who did not, and Mg concentrations in men who took/did not take Mg supplements were higher than in the corresponding subgroups of women.

### 3.7. Other Elements

#### Creatine Supplement

**Figure 4:**
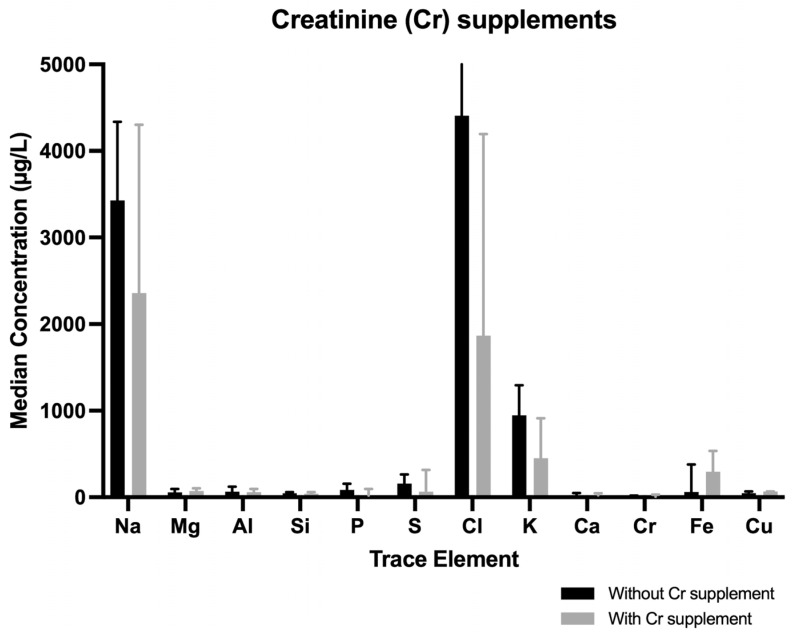
Median concentrations differences of trace elements when comparing participants (men and women) with and without Cr supplements. When creatinine supplements were consumed, S decreased and Fe increased.

Athletes who took creatine supplements (n = 7) showed higher but not statistically significant median concentrations of Fe or Mg in tears (Fe with vs. without supplement, 294.4 [109.3–535] µg/L vs. 60.3 [18.24–341.7] µg/L, *p* = 0.254; Mg with vs. without supplement, 73.325 [41.656–104.25] µg/L vs. 58.07 [38.5–95.14] µg/L, *p* = 0.694) (Figure 4).

### 3.8. Multivariate Analysis

**Figure 5:**
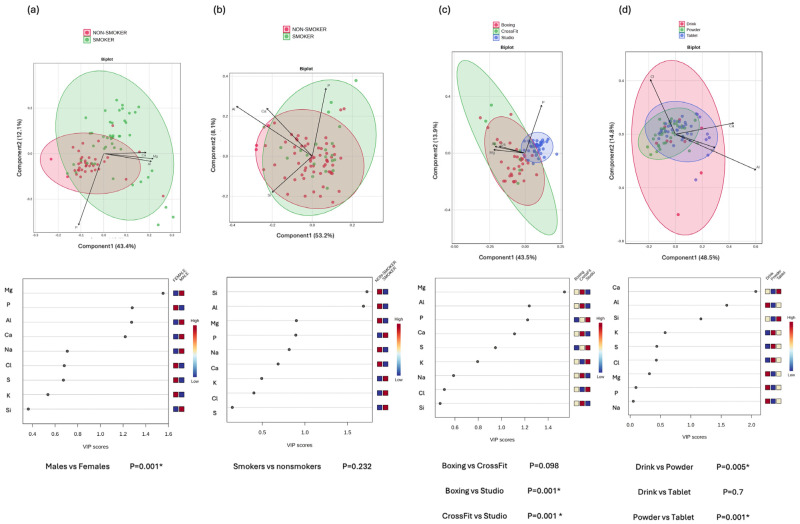
Multivariate analysis to identify subgroup-specific elemental profiles. Upper panels show Biplots of Partial Least Squares Discriminant Analysis (PLS-DA), illustrating the separation between the subgroups: (**a**) gender (male vs. female), (**b**) smoking status (smoker vs. non-smoker), (**c**) type of sport (boxing, CrossFit, studio-based exercise), and (**d**) supplement form (drink, powder, tablet). Each point represents an individual sample, and ellipses indicate 95% confidence intervals around group centroids. Arrows represent the top four features contributing to group separation. The arrow direction indicates the orientation of features, and the arrow length reflects the strength of each feature’s contribution. The lower panel displays the Variable Importance in Projection (VIP) scores for each subgroup comparison, ranking the importance of elemental features contributing to the observed separations. Elements with VIP scores > 1 are considered significant contributors to group differences. Heatmaps next to the VIP plots represent relative concentrations of the corresponding element. *p*-values beneath each plot were calculated using PERMANOVA. Statistically significant differences *p* < 0.05 (*) were observed between sexes, training types, and supplement forms. Notably, the studio group showed a distinct composition compared to both boxing and CrossFit, and the powder form differed significantly from both drink and tablet forms.

To investigate the differences in tear elemental composition based on gender, sport intensity, smoking status, and supplementation form, PLS-DA was performed (Figure 5) and the VIP scores derived from the PLS-DA model were used to identify the elements most responsible for group separation, with VIP values greater than 1 considered indicative of a strong contribution to the model.

Among the key elements identified, P (VIP = 1.28) was the only contributor higher in the female cluster, whereas Mg (VIP = 1.553), aluminum (Al) (VIP = 1.275), and calcium (Ca) (VIP = 1.219) contributed toward the male cluster. This directional separation corresponded to the observed differences in elemental concentrations, with P levels being higher in women and Mg and Al concentrations being higher in men.

In the analysis comparing smokers and non-smokers, only two elements showed high enough VIP scores: Silicon (Si) (VIP = 1.727) and Al (VIP = 1.687), both higher in smokers. However, overall, no significant separation was observed between the groups (*p* = 0.232).

When comparing types of physical activity, studio-based athletes exhibited significantly different elemental profiles compared to boxing (*p* = 0.001) and CrossFit athletes (*p* = 0.001), while no significant difference was detected between boxing and CrossFit groups (*p* = 0.098). Differences were mainly driven by Mg (VIP = 1.54), Al (VIP = 1.238), P (VIP = 1.224), and Ca (VIP = 1.128). Among these, P was the only element contributing predominantly to the separation toward the low-intensity group, while the other elements contributed toward the high-intensity groups.

Regarding supplementation forms, significant differences were found between drinks and powder users (*p* = 0.005) and between powder and tablet users (*p* = 0.001), but not between drink and tablet users (*p* = 0.7). In this comparison, Ca (VIP = 2.069), Al (VIP = 1.592), and Si (VIP = 1.166) were the main discriminatory elements. Powder supplementation is associated with lower concentrations of these elements.

## 4. Discussion

Concentrations of trace elements in tears have not been investigated in association with sports activities and supplement intake. In the present study, mean concentrations of trace elements were measured in tears of athletes using PIXE, and differences were found by gender, intensity of sports activity, and nutrient supplement intake. PIXE offers a sensitive approach for multi-elemental analysis in small-volume tear samples, and its feasibility in biological matrices has been demonstrated in prior studies, although further validation against established reference methods is warranted.

Although PIXE is more commonly used in materials science, its application to biological fluids has been validated by our group and others [14]. Using a dry matrix approach and careful calibration, the technique allows accurate quantification of multiple elements in very small tear volumes. The method’s reproducibility has been demonstrated in previous studies involving human tears and other biological samples, establishing it as a viable tool for non-invasive elemental analysis.

Studies have shown that self-administration of supplemental vitamins and minerals is common among athletes, particularly in those of higher socioeconomic status with healthier lifestyle habits, higher levels of education, and lower body mass indices. While prior reports suggest higher supplement use among female athletes [15], our cohort showed greater supplement use in men (65% vs. 42%), likely reflecting the male predominance in high-intensity sports. Income and education levels were unavailable, but all participants were attending private gyms, indicating a high economic status.

We found that, in contrast to earlier studies [15], 65% of the male athletes reported taking supplements compared to only 42% of the female athletes. Male athletes tend to use dietary supplements to increase muscle mass, and men engaged in very intense sports have a high consumption of specific sports supplements [15]. Women use supplements to be better physically prepared. In a study of 136 female amateur runners (median age 39 years), 33% took supplements during the period immediately preceding a running event [16].

Nevertheless, the effect of dietary supplements on athletic performance is controversial [17]. A study from Iran compared the effect of L-arginine with placebo in 56 male soccer players (mean age 21 years). The supplement takers had a significantly better VO_2_ max value [18]. However, a study of 150 students in northern Italy found that although 45% of the cohort reported using vitamins/minerals to improve their sports performance, there were no relevant differences between supplement consumers and non-consumers in terms of healthy behaviors, gender, and anthropometric characteristics other than a lower body mass index in the male supplement consumers [15].

The focus on Fe and Mg was based on their physiological relevance in athletic performance. Fe plays a central role in oxygen transport and energy metabolism, while magnesium contributes to muscle contraction, nerve function, and fatigue resistance. These elements are commonly included in dietary supplements used by athletes and were detectable in sufficient concentrations using the PIXE method. While we did not assess direct correlations with performance metrics, the aim of the current study was to evaluate the feasibility of using tear fluid as a non-invasive matrix to reflect systemic trace element variation in relation to supplement use and training intensity. This approach provides preliminary evidence that may support future studies linking elemental composition in tears to functional outcomes in sports settings.

Our study of trace elements in tears yielded significantly higher tear concentrations of phosphorus, potassium, and sulfur in women than men and in women participating in low-intensity sports compared to high-intensity sports. Men had higher concentrations of Fe and Mg than women with and without supplement use. Findings for Fe and Mg were further compared by type of sports activity, at three levels of intensity: CrossFit, boxing, and studio-based sports. It is also possible that differences in tear mineral content are influenced more by variations in dietary intake than by sport intensity alone. Athletes engaged in higher-intensity training may consume more food and supplements, which could contribute to the observed trends. This potential confounder limits the ability to draw definitive conclusions about the relationship between physical activity and tear element levels.

CrossFit is a high-intensity functional training method consisting of daily workouts. No dietary supplement recommendations exist for CrossFit that are supported by scientific evidence [2]. Studies conducted to date of effective strategies to improve performance and enhance adaptations and recovery had methodological shortcomings and yielded unclear findings [2].

Boxing is considered a moderate-high-intensity sport. It requires physical fitness, measured by anaerobic thresholds and maximal oxygen consumption, and upper-body muscular strength, measured by hand-grip strength [19]. A study from Italy of 214 boxers (88.4% male) showed that most took supplements, usually vitamins and mineral salts, to improve their nutritional status and energy level. Competing boxers consumed supplements recommended by coaches, whereas non-competitive boxers tended to consult a doctor [20].

While certain trends were observed—such as higher Mg and Fe levels in relation to supplement intake—many of these differences did not reach statistical significance. These findings should therefore be interpreted with caution and viewed as exploratory. The observed patterns may suggest underlying physiological relationships, but further studies with larger, more balanced cohorts are required to confirm these associations.

Low levels of magnesium can lead to muscle weakness and spasms [21]. Studies have shown that the amount of magnesium in both plasma and urine decreases after intense exercise. The more anaerobic the exercise, the greater the movement of magnesium from plasma to erythrocytes [22,23]. For example, marathon runners, who show significant decreases in serum and urine magnesium, perform better, with improved cardiovascular health, if they consume adequate amounts of unsaturated fat, iron, potassium, and magnesium [24]. In one randomized controlled trial of 30 healthy individuals aged 18–22 years, 4 weeks of magnesium supplementation were associated with an improvement in exercise performance, measured by the 20 m shuttle run test [25]. Our findings of higher Mg levels in men and high-intensity athletes are consistent with Mg’s role in muscle function and its mobilization during anaerobic exercise.

Fe and Mg are secreted in sweat [26]. Men were found to lose a notable amount of both minerals, in addition to sodium and phosphorus, in sweat during 16 continuous days of exposure to environmental temperatures of 100°F. In addition, sweat and urine magnesium concentrations increased with intense anaerobic exercise or competition [27]. Nevertheless, in the present study, Fe and Mg concentrations were still higher in men engaged in high-intensity sports than in men doing low-intensity studio-based sports, although dietary intake might also influence these levels.

Since most of our high-intensity athletes were men, we did not have enough data on high-intensity female athletes.

Fe is a functional component of oxygen transport and energy production in humans and therefore is a critically important micronutrient for sport and exercise performance [28]. We found that men had higher tear concentrations of Fe than women, and men attending boxing or CrossFit gyms had higher concentrations than men training in studio-based gyms. Even when women took Fe supplements, their Fe concentrations were lower than in men not taking supplements. The observed sex-related differences in tear element concentrations may reflect underlying physiological factors, including menstruation-related Fe loss, differences in muscle mass and metabolic demand, as well as the unequal distribution of high-intensity sports participation between men and women in our cohort.

A review by Alaunyte et al. [28] focused on studies investigating the effects of dietary Fe treatments on Fe status in female athletes. Female athletes were considered to be at a greater risk than male athletes of compromised Fe status, which may lead to Fe deficiency (with or without anemia) due to insufficient dietary Fe intake, menstruation, increased Fe losses associated with hemolysis, sweating, gastrointestinal bleeding and exercise-induced acute inflammation. Others reported that factors affecting the increased risk of Fe deficiency and related Fe deficiency anemia in female athletes included foot-strike induced hemolysis, insufficient dietary intake, increased Fe losses, and suppressed intestinal Fe absorption caused by inflammation [29]. Identifying and correcting Fe deficiency could have a significant impact on their performance and well-being [28].

Smoking has been previously associated with alterations in trace element metabolism, including reduced Fe absorption and increased oxidative stress. Only 25% of our cohort were smokers. In our cohort, smokers exhibited a trend toward lower Fe concentrations in tear fluid, which may reflect systemic effects of tobacco exposure. Although PIXE did not detect elevated levels of smoking-associated elements such as cadmium or chromium in tears, as was noted in previous studies [9], this trend is consistent with literature suggesting smoking-induced disruptions in Fe homeostasis. Additionally, our prior research on individuals exposed to wildfire smoke revealed similar elemental shifts, underscoring the influence of environmental particulates. Given PIXE’s high sensitivity to elements like aluminum, silicon, and titanium, we also considered potential confounding sources such as cosmetics and environmental exposure, and applied exclusion criteria accordingly to minimize contamination.

Smoking is known to decrease levels of vitamin C, which weakens the body’s ability to absorb Fe, leading to Fe deficiency anemia. Smoking also alters levels of vitamin B12 and folic acid, resulting in macrocytosis. In addition, anemia may be caused by oxidative stress, inflammation, bone marrow depression, and smoking-induced gastritis [30].

In a study by Satarug et al. [29] from Australia, smokers (all men) had an approximately two-fold higher cadmium body burden than non-smokers. Chronic exposure to low-level cadmium has been linked to osteoporosis, kidney dysfunction, diabetes-related renal complications, hypertension, and cancer.

Seven male athletes consumed creatine supplements. Our review of the literature yielded over 500 studies evaluating the effects of creatine supplementation on muscle physiology and/or exercise capacity in healthy populations, athletes, and patients with various diseases [31]. Although not all reported significant results, the preponderance of scientific evidence indicates that creatine supplementation appears to be a generally effective nutritional ergogenic aid for a variety of exercise tasks in several populations [31]. In our study, the consumption of creatine had no effect on the concentrations of trace elements in tears (only a trend to reduce S and elevate Fe).

To date, owing to technical difficulties, few studies have investigated tear composition, both in general and in terms of the concentrations of trace elements associated with sports activity. Several considerations were taken into account to estimate the element concentration in the samples in our study. One of the main challenges with liquid drop deposition on a paper substrate is the occurrence of chromatographic effects and evaporation processes. These processes arise as the liquid diffuses through the substrate by capillary forces. The characteristic ring formation at the edge is caused by the evaporation of liquid from the paper edge and replenishment with liquid from the inner (central) region due to capillary forces creating a concentration gradient. Therefore, we suggest that the tear spot on the Schirmer paper is homogeneous. The present study showed that PIXE may be applied for sensitive and specific analysis of even small tear samples without manipulation.

The lack of difference in some trace elements in tears between subjects who took or did not take supplements might be explained by an increased secretion of the supplements in urine or utilization of the elements during intensive activity. We did not compare tears levels to blood or urine levels. We assume levels in tears might be associated with urine secretion and also with dehydration level or electrolyte imbalance. These factors warrant further exploration.

The reported impact of age on diet quality is interesting [2,4]. The present study was limited to young adults, so no effect of age on tear elements could be demonstrated.

In addition to athletic populations, the use of PIXE for tear analysis may hold future potential for detecting disease-related trace element imbalances in older adults or individuals with chronic conditions. Preliminary findings from our ongoing work in Wilson’s disease suggest that copper can be reliably detected in tears, highlighting the feasibility of this method for early diagnosis or monitoring in metabolic, psychiatric, or systemic diseases.

An important limitation of this study is the absence of comparative analysis with other body fluids such as blood or urine. Without such data, the interpretation of tear element concentrations as biomarkers of systemic health remains speculative. Future prospective studies in a controlled athletic setting that will include concurrent tear, blood, urine, and sweat sampling should be performed to more definitively evaluate these associations.

Another limitation of the present study is the absence of a sedentary control group. Including non-athletic participants without supplement intake in future studies would help distinguish the specific contributions of physical activity and supplementation to trace element variations in tear fluid and may reveal subclinical deficiencies or accumulation patterns otherwise masked within athletic populations.

This study has several additional limitations, including its retrospective design and the unequal distribution of participants across subgroups, particularly the low number of women in high-intensity sports. These imbalances reflect real-world participation trends observed in the gyms where recruitment occurred. While this limits the statistical power and generalizability of some findings, the exploratory nature of the study and the use of a novel, non-invasive analytical method (PIXE) provide meaningful preliminary data. Future prospective studies with larger and more balanced cohorts are warranted to validate and expand upon these findings. Another limitation of the study is the lack of body composition and behavioral data, which are known to influence nutrient absorption and trace element bioavailability. As this was a minimally invasive, field-based study, we prioritized participant compliance and limited data collection to non-intrusive measures. Future prospective studies in clinical or controlled settings should incorporate detailed assessments of body composition to better contextualize tear-based elemental findings.

## 5. Conclusions

This study demonstrates the feasibility and potential utility of using PIXE to noninvasively measure trace elements in tear fluid, revealing significant differences in elemental composition based on gender, exercise intensity, and nutritional supplementation. These findings suggest that tear analysis, an easily accessible and minimally invasive method, may serve as a novel biomarker platform for monitoring systemic mineral status in athletes. The observed associations between Fe and Mg levels and sport intensity or supplementation indicate possible physiological or metabolic adaptations that merit further exploration. Notably, the discrepancies in elemental response between men and women highlight the need for sex-specific approaches in nutritional strategies for athletic populations. While preliminary, our results support the concept that tear composition reflects systemic exposures and may complement existing methods for monitoring nutritional status. Future prospective studies incorporating blood, urine, and sweat analysis are warranted to validate tears as a clinical tool and to determine whether tear-based biomarkers could inform personalized supplementation or training programs in both athletic and clinical populations. These findings open the door for the development of tear-based, noninvasive screening tools to monitor mineral status and nutritional adequacy in athletes. Such tools could support individualized supplementation plans, early detection of deficiencies, or monitoring of recovery and physiological stress. Furthermore, this approach may have broader applications in clinical settings, particularly for populations where frequent blood sampling is impractical.

## Figures and Tables

**Table 1:** Background data on 84 athletes participating in the study.

Characteristics	High Intensity		Low Intensity	
	Women (*n* = 5)	Men (*n* = 37)	Women (*n* = 33)	Men (*n* = 9)
Age (years), mean	35	34	30	36
Weight (kg), mean	63	83.5	63.5	82.5
Activity duration (h/week), mean	5	10	4	5
Type of sports, *n*				
CrossFit	1	6		
Boxing	4	31		
Studio-based exercise			33	9
Supplement intake, *n*				
Yes	2	25	14	6
No	3	12	19	3
Type of supplement				
Fe		10	5	1
Mg	1	19	8	
Zn	1	14	7	1
Protein/vitamins	2	22	9	4
Smoking				
Yes	1	7	9	4
No	4	30	24	5

Note: The table describes the questionnaire-derived data on demographics, intensity of sports activity, supplement intake, and smoking status The sum of specific supplement types exceeds the number of supplement users, as some participants reported taking more than one supplement (e.g., a multivitamin containing both Fe and Mg).

**Table 2:** Concentration of different elements in tears of 84 athletes.

Element	Median (µg/L)	IQRs Range	Excluded <LOD
Males			
Na	2804	1709.5–3913.0	2
Mg	59.07	38.8–99.7	10
Al	62.19	36.2–122.1	15
Si	46.97	23.7–61.6	1
P	28.135	10.2–92.9	5
S	123.75	24.7–194.3	3
Cl	3432.5	1802.5–4662.2	3
K	651.4	308.5–1016.0	1
Ca	26.405	18.4–44.6	7
Cr	12.05	5.5–22.1	28
Fe	159.3	86.4–546.1	21
Cu	56.435	34.4–67.9	25
Females			
Na	3789	2534.5–4482.0	5
Mg	44.47	23.4–94.5	25
Al	65.09	25.4–252.2	27
Si	45.92	32.6–53.5	1
P	122.8	78.3–197.0	1
S	240.95	159.1–320.9	4
Cl	5090.5	4234.7–6315.5	0
K	1117	860.6–1405	1
Ca	21.74	7.3–48.8	12
Cr	14.825	4.5–22.4	29
Fe	18.27	3.6–54.9	22
Cu	43.4	32.8–60.9	28

Note: Findings on PIXE analysis of the elemental concentrations of tears in the cohort. Cu levels were equal to the limit of detection. Zn levels were undetectable. IQRs, Interquartile ranges; LOD, limit of detection.

## Data Availability

Data described in the manuscript will be made available from the corresponding author upon reasonable request due to patient privacy.

## Abbreviations

The following abbreviations are used in this manuscript:

Fe	Iron
Mg	Magnesium
Al	Aluminum
Ca	Calcium
Si	Silicon
K	Potassium
S	Sulfur
P	Phosphorus
PIXE	Particle-induced X-ray
LOD	Limit of detection
PLS-DA	Partial Least Squares Discriminant Analysis
VIP	Variable Importance in Projection
PERMANOVA	Permutational Multivariate Analysis of Variance
